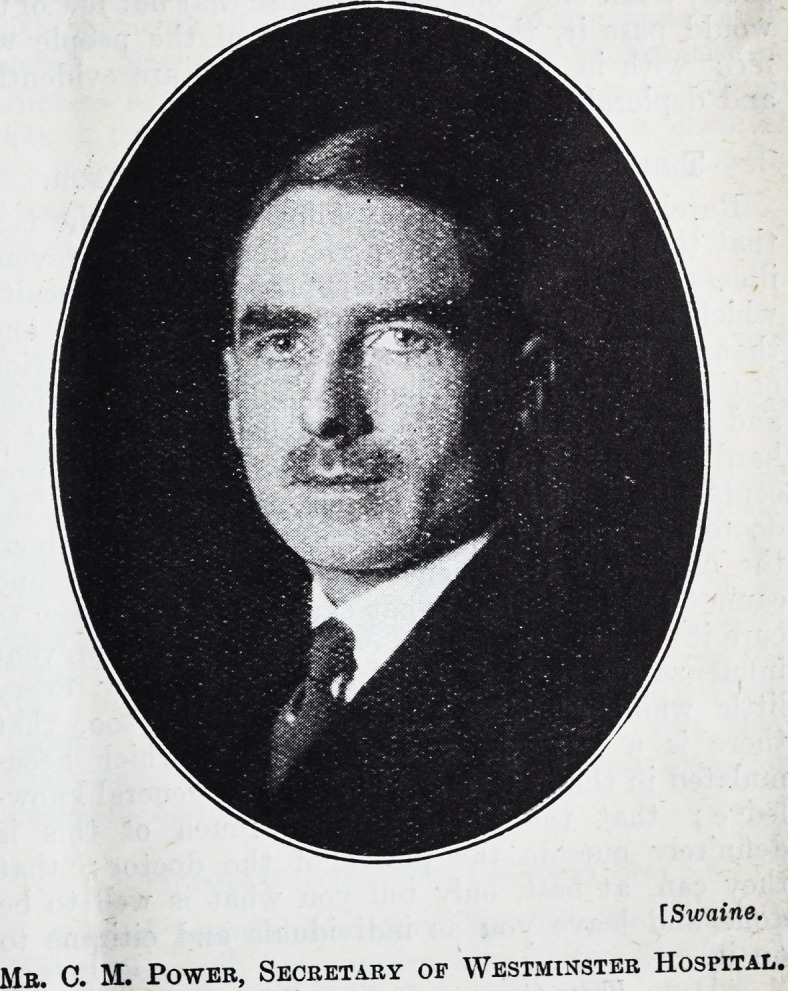# Hospital Men of Mark: Sir Edward Pearson and Mr. C. M. Power

**Published:** 1924-04

**Authors:** 


### HOSPITAL MEN OF MARK.

### SIR EDWARD PEARSON AND MR. CHARLES M. POWER.

THE world at large knows little of the self-sacrifice of men who, without any motive but sympathy for the sick poor, give unstintedly of their best to the administration of the Voluntary Hospitals. At Westminster Hospital, Sir Edward Pearson, although busily engaged as a Director of the great firm of S. Pearson & Sons, Ltd., finds time to carry out in the fullest possible way his duties as Chairman. Unanimously elected in 1921, in succession to the late Lord Glenconner, his remarkable memory, his genius for organisation, and his sound advice on all matters of policy have been of inestimable value to the Hospital. Sir Edward's sympathy with and interest in all matters connected with the nursing staff and daily routine have endeared him to all. Under his guidance this great Hospital dealing with over 17,000 sick men, women and children yearly is administered as economically as any Hospital in London. His example inspires everyone connected with the Institution, governors, doctors, nurses and students, to do their utmost whenever their help is called for, and all work together as one happy family.

[*Lafayette.*

SIR EDWARD PEARSON, CHAIRMAN OF WESTMINSTER HOSPITAL.

[*Swaine.*

MR. C. M. POWER, SECRETARY OF WESTMINSTER HOSPITAL.

In connection with the reconstruction of the Hospital, which is now in progress, Sir Edward Pearson has been able to give most valuable, technical and practical advice which has been the means, not only of saving expenditure, but of ensuring that the maximum of improvement and efficiency will be obtained for the money spent. Sir Edward is now working hard to raise the £50,000 necessary to defray the cost of the reconstruction, and to enable the Hospital to re-open its doors next yeat free from debt, and his name in this connection will no doubt, during the next six months, become familiar with those who have at heart the welfare of the sick poor.

Associated with Sir Edward Pearson is Mr. Charles M. Power, Secretary of the Hospital. Appointed in 1921 he had previously served in every administrative department of St. Bartholomew's Hospital. He was in France from October 1914 to May 1919, and was successively Company Commander, Adjutant, and Brigade Major. He won the Military Cross, and was mentioned in despatches, also receiving the Mons Medal and Star. The Secretary's life at Westminster Hospital is made pleasant because of the spelndid way in which committee, doctors, nursing staff and students, as well as the Ladies' Association and doctors' wives and friends, work together for the good of the Hospital and the benefit of the patients. Mr. Power is one of the many people who with intimate knowledge of Hospital co-administration desires to see better Hospital co-operation, not only in the work of relieving the sick poor, but also in the great problem of obtaining the necessary financial help for maintenance and for structural alterations which from time to time are essential if the Hospitals are to keep pace with the progress of medical science. It will not be forgotten that Mr. Power has contributed articles on the subject in THE HOSPITAL AND HEALTH REVIEW.

## Figures and Tables

**Figure f1:**
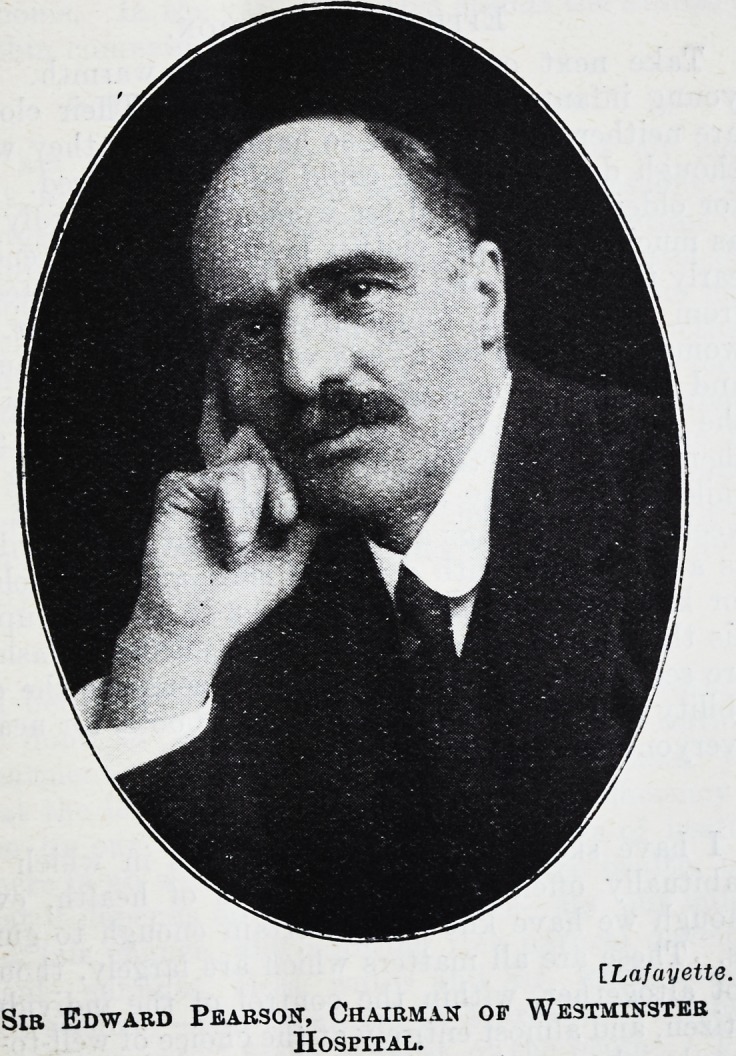


**Figure f2:**